# PathwayNexus: a tool for interactive metabolic data analysis

**DOI:** 10.1093/bioinformatics/btae310

**Published:** 2024-05-09

**Authors:** Philipp Eberhard, Martin Kern, Michael Aichem, Hanna Borlinghaus, Karsten Klein, Johannes Delp, Ilinca Suciu, Benjamin Moser, Daniel Dietrich, Marcel Leist, Falk Schreiber

**Affiliations:** Department of Computer and Information Science, University of Konstanz, Konstanz 78464, Germany; Department of Computer and Information Science, University of Konstanz, Konstanz 78464, Germany; Department of Computer and Information Science, University of Konstanz, Konstanz 78464, Germany; Department of Computer and Information Science, University of Konstanz, Konstanz 78464, Germany; Department of Computer and Information Science, University of Konstanz, Konstanz 78464, Germany; Department of In Vitro Toxicology and Biomedicine, University of Konstanz, Konstanz 78464, Germany; Department of In Vitro Toxicology and Biomedicine, University of Konstanz, Konstanz 78464, Germany; Department of Computer and Information Science, University of Konstanz, Konstanz 78464, Germany; Department of Human and Environmental Toxicology, University of Konstanz, Konstanz 78464, Germany; Department of In Vitro Toxicology and Biomedicine, University of Konstanz, Konstanz 78464, Germany; Department of Computer and Information Science, University of Konstanz, Konstanz 78464, Germany; Faculty of Information Technology, Monash University, Clayton, 3800, Australia

## Abstract

**Motivation:**

High-throughput omics methods increasingly result in large datasets including metabolomics data, which are often difficult to analyse.

**Results:**

To help researchers to handle and analyse those datasets by mapping and investigating metabolomics data of multiple sampling conditions (e.g. different time points or treatments) in the context of pathways, *PathwayNexus* has been developed, which presents the mapping results in a matrix format, allowing users to easily observe the relations between the compounds and the pathways. It also offers functionalities like ranking, sorting, clustering, pathway views, and further analytical tools. Its primary objective is to condense large sets of pathways into smaller, more relevant subsets that align with the specific interests of the user.

**Availability and implementation:**

The methodology presented here is implemented in *PathwayNexus*, an open-source add-on for Vanted available at www.cls.uni-konstanz.de/software/pathway-nexus.

**Contact:**

falk.schreiber@unikonstanz.de

**Supplementary information:**

Website: www.cls.uni-konstanz.de/software/pathway-nexus

## 1 Overview

Advancements in technology for high-throughput screenings lead to ever-increasing datasets in biochemical research, as exemplified by Sostare *et al.* (2022). Investigating this wealth of information requires effective approaches, and one such strategy involves defining pathways of biochemical reactions and associating the data with their corresponding positions in these pathways. Metabolic pathways—networks of reactants (metabolites) that are built over sequences of chemical reactions catalysed by enzymes—are crucial for comprehending the dynamic processes within living organisms. Given the large metabolomics datasets, it can be challenging to detect which pathways are affected by the experiment. In order to detect such a set of pathways, the data can be mapped on pathways followed by analysis methods that identify pathways of interest. In contrast to classical pathway analysis provided by most existing tools and online services, it is very beneficial to adopt more intuitive approaches, such as informative overviews, visually guided exploration, or the definition of new metabolite groups as pathways. We present a tool that provides such methods including an intuitive overview and interactive exploration of data-enriched pathways (pre- or self-defined). It is implemented as an add-on for Vanted (Rohn *et al.*, 2012), using Vanted’s visualization and database (DB) access functionalities.

There are many tools and online services available for data mapping and pathway enrichment. Examples include TabPath (de Moraes *et al.*, 2018), a web service that uses pathways for comparative genetics analyses and provides functionality to connect genomic and proteomic data to pathways from KEGG, and MetExplore (Cottret *et al.*, 2018), a web service for metabolic network curation and analysis that can map data from metabolomics experiments on metabolic pathways, supports pathway enrichment, and provides visualization of the mapping. However, to our knowledge, existing tools do not provide this combination of intuitive overviews, defining metabolite groups as new pathways and interactive hierarchical data investigations.

**Figure 1.**
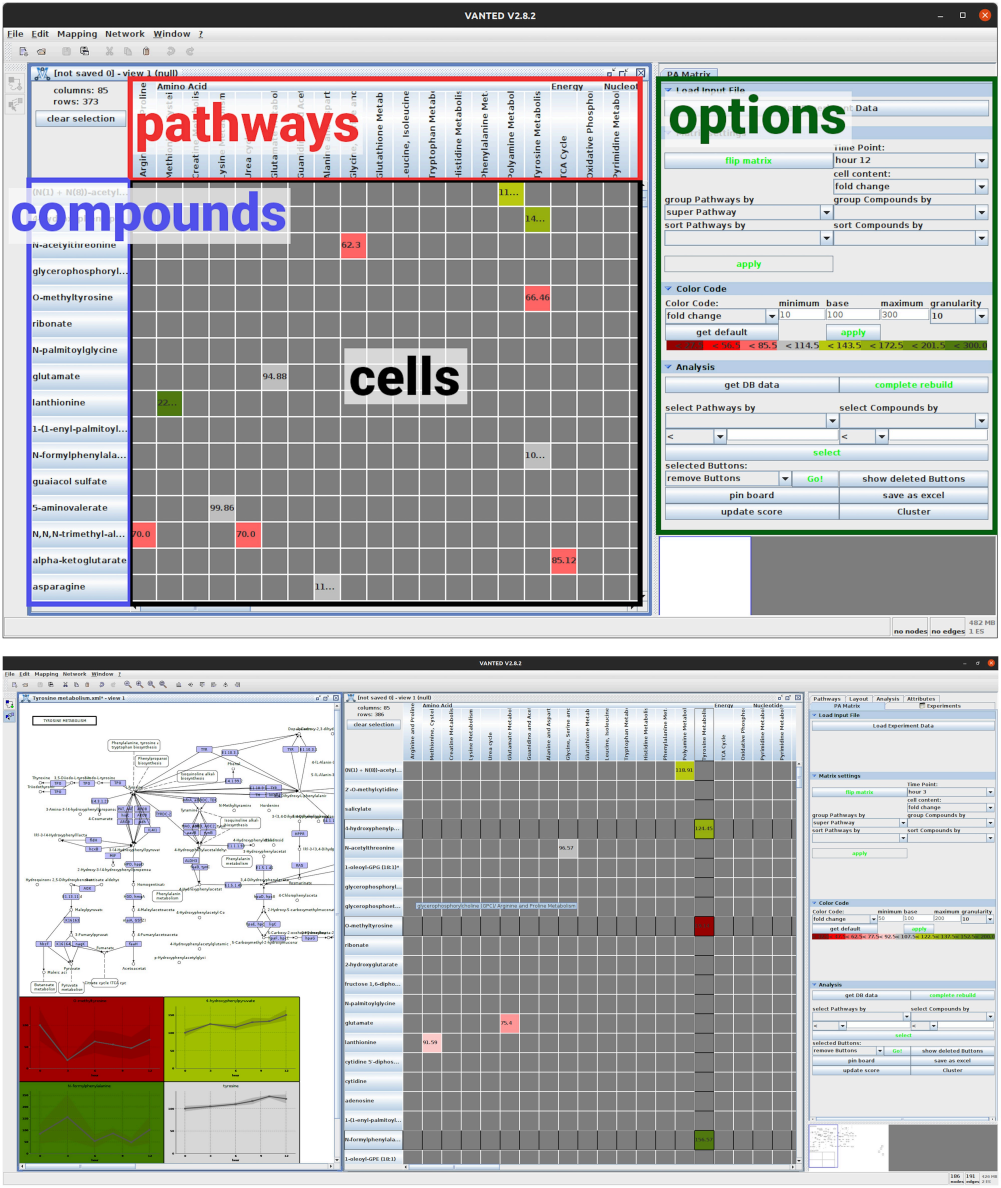
Screenshots of *PathwayNexus* with (top) its basic components highlighted. Red: pathways; blue: compounds; black: matrix cells; green: side bar containing display options and tools for analysis. (Bottom) an example use case also showing a pathway selected from the matrix.

Pathways can be created within Vanted (which allows to deviate from the classical pathways that often seem quite artificial) or downloaded from DBs such as KEGG, BioModelsDB, and others. Data are loaded as a spreadsheet that provides information about metabolites (or other entities that could be mapped on pathways), optional DB identifiers and experimental data assigned to metabolites (e.g. concentrations, fold changes, *P*-values, etc. over time or under different treatments). The user can add information for the mapping, which is then displayed in a matrix with substances and pathways on one axis each. The cells contain values shown with colour codes and information on the substances (see Fig. 1). *PathwayNexus* provides the following methods:

- Sorting pathways/compounds by properties of the data, e.g. sort compounds according to their fold change at a certain time point.
- Hiding pathways/compounds based on data properties, e.g. hide pathways that contain less than a certain amount of measured compounds.
- Defining new pathways or clusters of co-regulated metabolites.
- Introducing categories to group pathways/compounds.
- Assigning scores to pathways using various algorithms, e.g. amount of measured substances or MSEA (Metabolite Set Enrichment Analysis). This score can then be used for ranking or excluding pathways.
- Displaying selected compounds in a pin board as 2D plots of the data.
- Opening network views of pathways from the matrix.
- Clustering metabolites using a variety of algorithms.

These methods can reduce pathways to a selection of pathways that show patterns, which make them interesting for further analyses. The matrix format is generally effective for managing up to 150 pathways and several hundred metabolites, offering benefits in both visualization and computational processing. It is feasible to work with larger datasets, including thousands of metabolites; however, preparing such extensive datasets with several samples per metabolite can require a few minutes processing time when loading due to the significant computational work involved in initial data analysis.

## 2 Use case

We use metabolomics data from a proteasome inhibition experiment (Gutbier *et al.*, 2018): a model for post-mitotic neurons (Suciu *et al.*, 2023) was treated with the proteasome inhibitor MG132 (Lee and Goldberg, 1998). Proteasome inhibition is considered important in *Parkinson’s disease* (Schildknecht *et al.*, 2017). Downstream effects of proteasome inhibition by MG132 are loss of mitochondrial membrane potential, depletion of glutathione (GSH) content, increased levels of reactive oxygen species, and apoptosis. The add-on was used to establish a model for cellular processes in *Parkinson’s disease* where the data consist of metabolites and their concentrations at certain points over a period of time. The analysis yielded a set of metabolic pathways apparently affected by the underlying treatment, and the conclusions from this analysis offer insights into mechanisms behind the effects of MG132 (Suciu *et al.*, 2023).

Metabolic data were loaded into *PathwayNexus*. *P*-values were calculated from the concentration fold changes (corrected for false discovery rate) and used along with the biochemical names and DB IDs. One-hundred sixty-one metabolites were annotated with KEGG compound names. The pathways containing these compounds were then loaded from KEGG. *PathwayNexus* automatically generated a matrix representation from this mapping of metabolites onto pathways. Compounds and pathways are represented by rows and columns, respectively (Fig. 1). Coloured cells depict whether a compound is active in a pathway. Each pathway was assigned a *P*-value, calculated via MSEA, an adaption of gene set enrichment analysis (Mootha *et al.*, 2003). Pathways can be filtered by statistical criteria, which was done for all 188 pathways at every measured time point and yielded 40 significantly deregulated pathways (at one or more time points). Visual investigation and literature research revealed that 13 of them have a significant relevance for the underlying experiment. The other pathways are not present in human neurons or the metabolites supporting them were considered too unspecific/promiscuous. *PathwayNexus* was then used to generate network representations of these data-enriched pathways.

## 3 Future work

There are a couple of directions into which *PathwayNexus* could be extended in the future. More than one dataset could be supported in order to enable multi-omics data analyses for the same organism. Data and pathways for different organisms could be considered, using, e.g., two-and-a-half dimensional pathways visualization (pathways stacking) as in Brandes *et al.* (2004). As the matrix format in general is effective for managing up to 150 pathways and several hundred metabolites, for larger datasets, additional methods for scalability (such as further aggregation or filtering techniques) could be developed.

## Data Availability

The software including documentation and example data is available from www.cls.uni-konstanz.de/software/pathway-nexus. The source code can be obtained from github.com/LSI-UniKonstanz/pathway-nexus.
